# Bacterial Multidrug Efflux Pumps at the Frontline of Antimicrobial Resistance: An Overview

**DOI:** 10.3390/antibiotics11040520

**Published:** 2022-04-13

**Authors:** Lulu Huang, Cuirong Wu, Haijiao Gao, Chao Xu, Menghong Dai, Lingli Huang, Haihong Hao, Xu Wang, Guyue Cheng

**Affiliations:** Ministry of Agriculture Laboratory for Risk Assessment of Quality and Safety of Livestock and Poultry Products, Huazhong Agricultural University, Wuhan 430070, China; huanglu@webmail.hzau.edu.cn (L.H.); wcrvary@outlook.com (C.W.); haizhijiaozi_79@163.com (H.G.); litianzhi@webmail.hzau.edu.cn (C.X.); daimenghong@mail.hzau.edu.cn (M.D.); huanglingli@mail.hzau.edu.cn (L.H.); haohaihong@mail.hzau.edu.cn (H.H.); wangxu@mail.hzau.edu.cn (X.W.)

**Keywords:** antimicrobials, antibiotic residue detection, biofilm, efflux pumps, multidrug resistance

## Abstract

Multidrug efflux pumps function at the frontline to protect bacteria against antimicrobials by decreasing the intracellular concentration of drugs. This protective barrier consists of a series of transporter proteins, which are located in the bacterial cell membrane and periplasm and remove diverse extraneous substrates, including antimicrobials, organic solvents, toxic heavy metals, etc., from bacterial cells. This review systematically and comprehensively summarizes the functions of multiple efflux pumps families and discusses their potential applications. The biological functions of efflux pumps including their promotion of multidrug resistance, biofilm formation, quorum sensing, and survival and pathogenicity of bacteria are elucidated. The potential applications of efflux pump-related genes/proteins for the detection of antibiotic residues and antimicrobial resistance are also analyzed. Last but not least, efflux pump inhibitors, especially those of plant origin, are discussed.

## 1. Introduction

Bacteria evolve mechanisms of defense against harmful external substances that threaten their survival. Transporters are located on the bacterial cell membrane and play important roles in decreasing the concentration of endogenous and extraneous substances and promoting the growth of bacteria. It should be noticed that some transporters are also involved in the biodegradation of environmental toxic compounds [1]. Therefore, bacterial efflux pumps not only work as a functional transporter but also defend from environmental stress to maintain bacteria survival. Although some efflux pumps have specific substrates, some transporters can pump out multiple different kinds of antimicrobials, inducing multidrug resistance [2]. Sometimes, the extrusion of organic solvents or other substrates leads to the overexpression of transporters, thus creating co-selection of antimicrobial resistance features [3]. The overexpression of efflux pumps would also impact bacterial pathogenicity, involving biofilms and quorum sensing (QS) [4,5]. The efflux pumps export not only antimicrobials but also virulence determinants, including adhesins, toxins, or other proteins that are important for colonization in host cells [6]. 

Many studies have described efflux pumps-mediated resistance mechanisms, and more and more new efflux transporters and related proteins have been discovered. In recent years, additional functions of transporters were investigated, which involved bacterial virulence and self-protection against environmental pollutants. Nevertheless, the exact mechanisms and active domains of efflux pump transporters have not been clearly elucidated. There are many influencing factors on the inner and outer bacterial membrane, stimulating pump activity and promoting structure alterations of the transporters in the fluid membrane environment.

This article briefly summarizes the functions of efflux pumps in common Gram-positive and Gram-negative bacteria. Applications of efflux pump-related regulator proteins for detecting antimicrobial resistance and antibiotic residues, as well as newly discovered plant-derived efflux pump inhibitors, are discussed.

## 2. Functions of Efflux Pumps

Efflux transporters are mainly grouped into the following superfamilies: ATP-Binding Cassette (ABC) superfamily, Multidrug and Toxic Compound Extrusion (MATE) superfamily, Major Facilitator Superfamily (MFS), Resistance Nodulation and Cell Division (RND) superfamily, and Small Multidrug Resistance (SMR) superfamily. In 2015, a novel transporter family was identified, known as the Proteobacterial Antimicrobial Compound Efflux (PACE) superfamily [7]. The discovery and identification of bacterial multidrug-resistant (MDR) efflux pumps is still ongoing [8]. One of the differences among efflux pumps is their source of energy. The ABC family members function as efflux pumps through the hydrolysis of ATP for energy supply [9], while other transporters including the MATE, MFS, RND, and SMR superfamilies utilize the proton-motive force provided by H^+^ or the electrochemical gradient of Na^+^ to supply energy and then extrude multiple compounds [10]. In addition, there are also differences in the composition of efflux transporters. The RND-type multidrug efflux proteins use a tripartite complex as the structural basis of the pump, including an outer-membrane canal protein (OMP), an inner-membrane transporter, and a membrane fusion protein (MFP) that connect the above two components to pump out drugs and other harmful compounds [11,12]. 

### 2.1. Efflux Pump-Mediated Antimicrobial Resistance

The resistance mechanisms to antimicrobials are based on changes in drug targets, structural modifications or degradation of drugs, decreased permeability of outer membrane proteins to prevent the drugs from entering the cells, and enhanced efflux transporters to reduce intracellular drug concentrations. It was generally believed that efflux pumps and outer membrane proteins did not have a synergistic effect on reducing intracellular drug concentrations. However, a recent study found an interaction between active efflux pumps and the permeability barrier of the outer membrane in Burkholderia thailandensis [13]. In fact, the overexpression of efflux pumps plays an essential role in the acquisition of antimicrobial resistance, even of multidrug resistance. Understanding the molecular structure of efflux pumps and their crucial drug binding sites is critical for the development of efflux pump inhibitors. The structures of different efflux pump families have been reviewed in detail and will be briefly discussed below [14,15]. In the following, the main focus is on the effects of efflux pumps on biofilm formation, bacterial virulence and invasion, and oxidative stress responses, in addition to the acquisition of antimicrobial resistance.

#### 2.1.1. ABC Superfamily

In Gram-negative microorganisms, the most extensively studied ABC-type transporters is the tripartite complexes MacAB-TolC efflux pump, which actively extrudes substrates including macrolides and polypeptide virulence factors powered by the ATPase MacB and participates in the secretion of enterotoxin TⅡ in *Escherichia coli* [16,17,18]. Rough-core lipopolysaccharide (LPS) or similar glycolipids is also regarded as a physiological substrate of the MacAB-TolC pump [18].The inner membrane protein MacB works as a homodimer complex that contains an N-terminal nucleotide binding domain that binds ATP and a C-terminal cytoplasmic tail [18]. The membrane fusion protein MacA binds the lipopolysaccharide core specifically and is activated by ATPase [18]. The tripartite-complex structure composed of the outer membrane protein TolC, the inner membrane protein MacB, and the periplasmic protein MacA provides a structural site for substrate transport [17]. The latest research shows that the lack of the MacAB efflux pump in *Serratia marcescens* increases the susceptibility to aminoglycosides and polymyxins, decreases the swimming motility and the ability of biofilm formation, even causes the loss of the ability to cope with superoxide stress [19]. In addition, MacAB also confers resistance to several penicillin-type antibiotics and As(III) in *Agrobacterium tumefaciens* 5A [20]. 

In Gram-positive microorganisms, the ABC transporter consists of a single transmembrane protein, such as EfrAB in *Enterococcus faecalis*, LmrA in *Lactococcus lactis* [21], Msr in *Streptococcus*, and PatA/B in *Streptococcus pneumoniae* [22,23]. The MDR pump EfrAB is a heterodimeric transporter that extrudes gentamicin, streptomycin, and chloramphenicol [24]. The expression of EfrAB is highly induced by sub-inhibitory concentration(sub-MIC) of these antibiotics and down-regulated by sub-MIC of EDTA (3 mM) [24]. The LmrA protein functions as a homodimer, comprising a nucleotide-binding domain and six alpha-helix transmembrane domains, which primarily recognize and transport macrolides and lincosamides [25]. The Msr protein harbors two nucleotide-binding domains with no transmembrane domain, conferring resistance to macrolides in *Streptococcus* and cooperating with the Mef transport family [26,27]. Nearly the whole family of hydrophilic fluoroquinolones, including ciprofloxacin and norfloxacin, is the substrate of PatA/B efflux pumps [28].

#### 2.1.2. MATE Superfamily

All members of the MATE family present 12 alpha-helical transmembrane regions powered by electrochemical ion gradients [29]. MATE transporters are classified into NorM, DinF (DNA damage-inducible protein F), and eukaryotic subfamilies according to amino-acid sequence similarity [30]. The substrates of MATE family transporters are diverse and have different chemical structures, but fluoroquinolones are the substrates recognized by almost all transporters [31].

Among the Gram-negative bacteria, the most studied pump is the NorM efflux pump in *Neisseria gonorrhoeae* and *Vibrio cholera* [32]. The NorM efflux pump exports substrates including antimicrobial cationic compounds (quaternary ammonium compounds) and antimicrobials such as ciprofloxacin and solithromycin in *N. gonorrhoeae* [33]. NorM has the ability to extrude intracellular reactive oxygen species, therefore reducing the damage of oxidative stress [34]. Mutations of the DinF transport system confer increased susceptibility to moxifloxacin, ciprofloxacin, and levofloxacin in *pneumococci* [35]. The competence-induced protein A is encoded by *cinA*, *recA*, and *dinF*, which form an operon that is induced by quinolones through the SOS response [35].

#### 2.1.3. MFS Superfamily

The MFS family is the largest characterized family of transporters, especially in Gram-positive microorganisms. Most members of this superfamily are MDR transporters and are constituted by 12 or 14 transmembrane segments [29]. The pumps Lde in *Listeria monocytogenes* [36] and NorA in *Staphylococcus aureus* [37] specifically extrude hydrophilic fluoroquinolones (e.g., ciprofloxacin and norfloxacin), while Mef in *S. pneumoniae* pumps out macrolides [38]. It has been found that the transcriptional start of *mef(E*)*/mel* genes locates in the *mef(E*)*/mel* promoter, and the attenuation of transcription will regulate *mef*-mediated macrolide resistance [39]. In addition, Msr of ABC family and Mef of MFS family enhanced macrolides extrusion synergistically, which increased resistance to 14-, 15-member ring macrolides [40].

In addition to extruding drugs, MFS transporter proteins also play important roles in other biological pathways. For example, MdrT and MdrM contribute to promoting the host immune response by activating IFN-β production of the type I interferon response and to maintaining cell wall stability [41]. Tet38, a chromosomally encoded MFS efflux pump, has an impact on multiple steps of the host cell invasion process of *S. aureus*, including adhesion, internalization, and trafficking in epithelial cells, and the subsequent step of the *S. aureus* infection in epithelial cells including bacterial viability and trafficking in phagolysosomes [41]. The AbaQ MFS transporter is also involved in *Acinetobacter baumannii* motility and virulence, as the loss of the *abaQ* gene decreases bacterial motility and virulence [41]. Additionally, the inactivation of genes encoding the RND, MATE, SMR, and ABC efflux pumps also reduces bacterial motility and virulence in comparison with the parental strain [42]. 

#### 2.1.4. RND Superfamily

RND efflux pumps are generally present in Gram-negative bacteria, which expel an extensive spectrum of antibiotics and toxic compounds They include AcrAB-TolC in *E. coli*, AdeABC in *A. baumannii* [43], CmeABC in *Campylobacter jejuni* [44], MexAB-OprM in *Pseudomonas aeruginosa* [45], MtrCDE in *N. gonorrhea* [46], OqxAB in *Klebsiella pneumoniae* and *Salmonella enterica* [47,48], SmeABC in *Stenotrophomonas maltophilia*, and TtgABC in *Pseudomonas putida* [49,50]. The Inner transporters such as AcrB, AdeB, CmeB, MexB, TtgB, SmeB, and MtrD are responsible for specific substrate binding and the transportation of different classes of drugs, playing vital roles in clinically relevant resistance [51]. For instance, mutations of the *acrB* gene would cause the failure of ciprofloxacin therapy [52]. The expression of the above-mentioned efflux pumps is also regulated by transcriptional regulatory proteins belonging to the TetR family, including AcrR [53], CmeR [54], NalC/NalD [55,56], TtgR [57], SmeT [58], and MtrR [59], as well as MexR belonging to MarR family [60]. Table 1 shows the efflux pumps regulated by the TetR family and summarizes the residues that have been proved to be important for the binding of the activating molecule by mutational analysis. Amino acid residues in efflux pumps may be important sites for substrates binding, and the substitution of amino acid residues may affect the affinity of the substrates. For example, Val 141, Phe 626, Tyr 330, and Phe 180 in the substrate-binding domain of the OqxB transporter protein are crucial for binding and transporting different substrates [61]. Additionally, mutations of important amino acid residues are associated with efflux pump-mediated multidrug resistance. The latest research shows that changing the glycine residue 311 to the acidic amino acid glutamic acid (G311E) in the MATE family protein DTX6 markedly increases Arabidopsis plants’ resistance to paraquat and diquat [62].

The multidrug resistance efflux pumps AcrAB-TolC, MexAB-OprM, CmeABC, MtrCDE of the RND family are involved in the survival and pathogenicity of bacteria [6]. Several studies have found that AcrAB-tolC efflux pump in *S. Typhimurium* and *E. coli* affect bacterial adhesion and invasion in host cells and colonization and persistence in animals [6]. Bacteria produce pore-forming toxins, degrading enzymes, and virulence factors of the secretory system to exert their pathogenicity and evade the attack from the host immune system. The outer membrane protein TolC can transport not only drugs but also hemolysin and colicin V [63,64]. 

Heavy metal ions are poisonous or inhibit the growth and survival of bacteria at specific concentrations. Due to the widespread application of heavy metal ions in antimicrobials such as antiseptic disinfectants, bacteria have to take measures to deal with the stress of heavy metals. In *E. coli*, the RND superfamily plays an important role in the resistance to antibiotics and heavy metals. There are seven known RND proteins; AcrB, AcrD, AcrF, MdtB, MdtC, and YhiV are multidrug efflux pumps of the hydrophobic and amphiphilic efflux RND (HAE-RND) protein family, while CusA is a member of the heavy-metal efflux RND (HME-RND) family that exports Cu(I) and Ag(I) [69]. CusA together with CusB membrane fusion protein and CusC channel protein constitute the complete tripartite CusCBA efflux complexes [70]. Long et al. predicted the mechanisms of metal ions export [70]. Firstly, Cu(I) is combined with the chaperone CusF and then is delivered to three-methionine metal-binding sites (M49, M64, and M66) at the long N-terminal tail of CusB; secondly, the metal ion is transferred to the three-methionine cluster (M573, M623, and M672) inside the periplasmic cleft of CusA and then is released into the central funnel of CusA; finally, the metal ion enters the CusC channel for its final extrusion [70]. Bacteria protect themselves from toxic components of organic pollutants by efflux pump biodegradation. TtgABC, a tripartite RND efflux pump, confers toluene tolerance in *P. putida* [71].

The stress caused by the abuse of antimicrobials increases the occurrence of functional mutations in the RND family, possibly enhancing its efflux function. The substrate specificity of efflux pumps correlates with differences of amino acid residues in the drug-binding pocket, and bacteria become less sensitive to antimicrobials after the substitution of these amino acid residues. Numerous examples of mutations in the RND efflux pump which have been identified from clinical and environmental isolates and laboratory-evolved strains affect antimicrobial resistance and are fully described in this article [72]. The acquisition of a functional mutation in an efflux pump may be an adaptive response of bacteria to antimicrobials and undoubtedly increases difficulties in the clinical treatment of bacterial infections.

#### 2.1.5. SMR Superfamily

The SMR family members consist of short polypeptide (100–150 amino acids) and span the cytoplasmic membrane as four transmembrane α-helices [73]. Those proteins with short hydrophilic loops permit to solubilization a broad spectrum of drugs, such as disinfecting agents including quaternary ammonium compounds, toxic lipophilic compounds including DNA intercalating dyes, toxic metabolites including nicotine intermediates, and polyamine compounds like spermidine [73,74]. In Gram-negative bacteria, the AbeS in *A. baumannii* transports sterilant ethidium, acriflavine, and benzalkonium, which also significantly increase resistance to amikacin [75,76]. The clinical strains of *K. pneumoniae* possesses the activated pump KpnEF and resists to benzalkonium chloride, chlorhexidine, and some other antiseptics [77]. The EmrE protein, present in both *E. coli* and *P. aeruginosa*, recognizes and mediates the extrusion of toxic polyaromatic compounds [78,79]. Specifically, the Qac protein is associated with resistance to some antiseptics and antibiotics [80]. The *qacA/B* genes is more frequently observed in *S. aureus* and *E. faecalis*, while the *qacE* gene is widely spread in *Enterobacteriaceae* and *Pseudomonas* spp. [80]. 

Genes encoding SMR proteins often occur on multidrug resistance plasmids and mobile genetic elements called integrons, which increase the risk of horizontal transmission of resistance [74]. Overexpression of efflux pumps induced by the exposure to QAC facilitates the horizontal transfer of mobile genetic elements carrying FQ resistance determinants (*qnr*, *aac(60)-Ib-cr*, *oqxAB*, *qepAB*) in Class 1 integrons (*QacED1*) [81]. Co-transfer of disinfectant-resistant genes and antibiotics-resistant genes among different species seriously influences the bactericidal effects of disinfectants and antibiotics.

#### 2.1.6. PACE Superfamily

The PACE transporter family has been identified in the last 5 years, and the Acel protein in *A. baumannii* is the first transporter protein found in the PACE family [82]. This transporter contributes to extruding biosynthetic biocides (e.g., chlorhexidine, acriflavine, proflavine, dequalinium, and benzalkonium) [7]. It has been found that genomes encoding the PACE transporter family are highly conserved among bacterial species, which suggests that PACE transporter-related genes are acquired perpendicularly and maintain their characteristic in host species [83].

AceI (Acinetobacter chlorhexidine efflux protein I) is similar to members of the SMR family in size and in the predicted secondary structure. AceI contains two tandem bacterial transmembrane pairs. BTP-domain proteins homologous to AceI have also been discovered in many pathogens such as *Burkholderia*, *Enterobacter*, *Klebsiella*, *Pseudomonas*, and *Salmonella* species [83,84,85]. The structure of the AceI protein is in equilibrium between a monomer and a dimer. Increases in chlorhexidine concentration and pH promote the formation of an acetylated dimer, and the binding of chlorhexidine to the transcriptional protein AceR increases *aceI* transcription, thus extruding chlorhexidine [82].

Table 2 and Table 3 summarize the major efflux pumps and their specific substrates in Gram-positive and Gram-negative bacteria, respectively.

### 2.2. Efflux Pumps Affect Biofilm Formation and Quorum Sensing (QS)

Biofilms, a microbial community attached to a surface, contribute to bacterial resistance and tolerance. Interestingly, the function of biofilms is directly or indirectly influenced by efflux pumps [94,95]. For instance, sub-inhibitory concentration of tigecycline directly restrain biofilm formation by downregulating the *adeG* efflux gene in *A. baumannii* [96]. QS is formed among cells, promotes the mutual communication of cells, and participates in bacterial activities through signal transduction. QS cooperation with biofilms improves the viability of bacteria by sensing changes in environmental signals. For example, the MexAB-OprM efflux pump extrudes acylated homoserine lactones with the contribution of QS, and overexpression of the Mex pump in *P. aeruginosa* results in the release of QS signals [6]. It has been found that QS among bacteria will be impeded if efflux pumps activity is hindered by inhibitors [97]. Similarly, the proper concentration of efflux pump inhibitors prevents biofilm formation, as observed for inhibitors of the NorA efflux pump in *S. aureus* [98].

The proteomic profiles of two *A. baumannii* strains grown in planktonic stationary phase or in mature solid–liquid biofilm were compared using proteomics, and the MacAB-TolC efflux pump was found to play an essential role in biofilm formation [99]. The efflux pump, which helps pathogen adapt to bad conditions occurring in mature biofilms, is involved in envelope stress responses that maintain membrane rigidity and confer resistance to high osmotic stress [99]. The MFS transporter proteins Pmt and AbaF are involved in *A. baumannii* biofilm formation. Pmt extrudes extracellular DNA and plays an essential role in forming the biofilm structure, while AbaF releases biofilm materials [41].

The RND-type MDR efflux system is well studied. It has been found that a number of RND transporters contribute to alterations of the membrane involved in bacterial functions, especially biofilm formation [100]. This was shown for *A. baumannii* efflux pump AdeB and its regulatory protein AdeRS. Deletion of *adeAB* genes or inhibition of the expression of these genes leads to the reduction or prevention of biofilm formation and of the QS system [101,102]. There is a positive correlation between biofilm formation and the mRNA levels of the efflux pump genes *adeB*, *adeG*, and *adeI*, which is altered by sub-MICs of colistin or polymyxin B [103]. The pump MexGHI of *P. aeruginosa* transport phenazine, which is required for biofilm morphogenesis [104]. The QS system has been shown to regulate the expression of RND efflux pumps. Conversely, the RND transporters can also influence QS via translocation of quorum signals [105]. It was shown that virulence and quorum sensing molecules of *P. aeruginosa* would be lost and reduced when the efflux gene *mexI* is mutated [106]. The ABC-type efflux system also plays a role in resistance to antifungal agents in fungi, particularly in *Candida* species. Similar to the RND pumps, it is involved in the secretion of quorum-sensing molecules and affects biofilms’ behavior [107].

## 3. Regulation of Efflux Pumps and Application of Efflux Pump-Related Genes/Proteins

### 3.1. Regulation Mechanisms of Efflux Pumps

In addition to exposure to antimicrobials or disinfectants, efflux pump expression is also regulated by a variety of regulatory systems and proteins. Currently, single regulatory proteins for the MDR efflux pump are mainly classified into four categories, i.e., AraC, MarR, MerR, and TetR [108,109,110]. These regulatory proteins contain DNA-binding domains and ligand-binding domains. For instance, the TetR family is a substrate-dependent transcriptional repressor and controls the expression of the efflux-related *tet* genes, which confer resistance to tetracyclines [111]. FepA is a novel type of MATE efflux pump, whose overexpression results in resistance to fluoroquinolones. It is regulated by FepR, a TetR-type repressor, which increases the MICs of norfloxacin and ciprofloxacin in *L. monocytogenes* [67]. The expression of the AceI protein, a transporter protein of the PACE family, is regulated by the LysR-type transcriptional regulator AceR [82]. There are extensively studies reporting that the transcriptional regulatory protein MepR, a MarR-type repressor, inactivates the MepA protein, whose substrates include biocides, fluoroquinolones, and tigecycline [92,93]. These regulators primarily respond to several types of signals including superoxide and peroxide (e.g., H_2_O_2_) [112], antimicrobials (e.g., antibiotics), toxic reagents (e.g., methylglyoxal), and biocides (e.g., triclosan) [113,114].

Another type of efflux pumps regulation is based on the two-component regulatory system, consisting of a cognate response regulator and a sensor histidine protein kinase, such as SoxRS, AdeRS, BaeRS, SmeRS, MacRS, ArlRS, EvgSA, BaeSR, CpxAR [115,116,117,118,119,120,121]. Each sensor detects a specific intracellular signal (e.g., antibiotics) and then induces histidine auto-phosphorylation, thus transferring the phosphate group to the proper aspartic acid and activating the response regulator [122]. For instance, it is known that AcrAB in *E. coli* is controlled by a series of different regulatory systems, including MarA, Rob, SoxS, AcrR, EnvR, MprA, PhoP, RpoE [123]. These regulatory systems are activated by external environmental signals, such as pH, the concentrations of antimicrobials, divalent metal ions, organic solvents, the growth phase, and oxidative stress [123].

### 3.2. Determination of Antimicrobial Resistance Based on Efflux Pump Gene Expression

Microorganisms harbor MDR efflux pumps resulting in inherent or acquired resistance to antimicrobial agents. The resistance-related genes encode the constituent proteins or regulatory proteins of efflux pumps, which function as the first line of defense against drugs, maintaining the survival of the bacteria [124]. Many efflux pump genes can be used for the rapid detection of antimicrobial resistance, which can be efficiently verified by PCR identification and MIC determination. The RND family of efflux pumps recognizes a large number of substrates, extruding the majority of drugs and increasing antimicrobial resistance. Examples are the Acr pump in *E. coli*, the Ade pump in *A. baumannii*, and the Mex pump in *P. aeruginosa* [76,125]. According to whether the efflux gene is expressed or not, we can determine antibiotic resistance and guide clinical therapy [126]. For instance, in the carbapenem-resistant *A. baumannii* isolate, the expression of the efflux genes *adeB, adeG*, and *adeJ* was increased by different folds [127]. Similarly, a stimulated expression of *adeB* and *adeJ* was also found in bacteria resistant to tetracyclines [128]. However, there is a slight relationship between substrates concentration and velocity of maximal transportation. For example, although cefaloridine can be strongly excreted by AcrB, it still possesses antibacterial activity in the presence of AcrB, which explains that the effective antibacterial dose of cefaloridine is much lower than the concentration required for the efflux [129].

In addition, some single-substrate efflux pumps are associated with high level of resistance and MICs. These single-substrate efflux proteins include the macrolide-specific efflux pumps MacAB in *E. coli* and Mef in *S. pneumoniae* [38], the hydrophilic fluoroquinolones efflux pump OqxAB in *E. coli* and *S. enterica* [130], the tetracycline-mediated efflux pump TetA/TetO in *E. coli* [131], etc.

### 3.3. Detection of Antibiotic Residues Based on Efflux Pump Proteins

Presently, efflux pump-related proteins are still under research for antibiotic residue detection, and only two regulatory proteins are used for the screening of antibiotics residues, including TetR and TtgR (belongs to TetR family). Receptor proteins involved in gene transcriptional regulation have been proven to be a powerful tool for detecting low antibiotic concentrations [132]. Hyerim et al. established a bioreporter method based on TetR and *tetR* promoter to detect doxycycline, using a green fluorescence protein gene as reporter gene, and 5nM doxycycline would induce high expression levels of green fluorescence protein [133]. Weber et al. developed an in vitro indirect enzyme-linked immunosorbent assay using TetR-*tetO* to accurately and rapidly detect tetracycline antibiotics, with the detection limits of doxycycline and tetracycline of 0.1 and 1.9 ng/mL, respectively [134]. Moreover, Espinosa-Urgel et al. established a novel microbial biosensor based on TtgR to detect drug concentrations around 22 µM with high fluorescence intensity [135]. It was demonstrated that green fluorescence protein-fused TtgR, a TtgABC efflux pump transcription regulator in *P. putida*, is most sensitive to ceftazidime, ciprofloxacin, and tetracyclines [135].

## 4. Efflux Pump Inhibitors (EPIs) in Antimicrobial Therapy

Efflux pumps act as a new target for antimicrobial combination therapy, enabling synthetic or plant-derived EPIs to assist antibiotics therapy against bacterial infections [136,137]. Multi-drug resistance is largely mediated by efflux pumps; hence, the development of efflux pump inhibitors is necessary to curb antimicrobial resistance. There are various ways to block or bypass the action of the efflux pumps, including decreasing the binding affinity of antibiotics to the transporter by modifying the drug’s chemical structure, increasing the permeability of the outer membrane to increase the intracellular drug concentrations, inhibiting or knocking out efflux pump-related genes, impairing the ATP energy supply, or designing substances that can compete with antimicrobials for the action site of efflux pumps to competitively inhibit the efflux activity [138].

A number of inhibitors have been discovered by computational analysis or artificial extraction from plants. Through high-throughput virtual screening of natural compound collections against NorM—a MATE transporter from *N. gonorrhea*—authors found that Terminalia chebula, a compound from an Indian medicinal plant, destroyed the binding of Na^+^ and ligands and turned the NorM transporter into a closed state [139]. Phenylalanyl–arginine β-naphthylamide, an inhibitor of AcrB in *E. coli*, has been shown to inhibit the extrusion of drugs via binding to the hydrophobic pocket of AcrB [140]. It can be combined with carolacton, a secondary metabolite in myxobacteria, for potential use in antimicrobial chemotherapy against AcrAB-TolC [141]. Tannic acid also acts as an inhibitor of *S. aureus* multidrug efflux pump, Tet, Msr, and others [142]. It has been found to significantly reduce the MIC of drugs such as tetracycline and erythromycin [142]. However, EPIs combined with antibiotics to assist MDR therapy in the clinic is an obviously potential challenge, depending on the intrinsic permeability properties of the bacterial outer membranes. Yang et al. demonstrated that the coupled use of tobramycin and EPI promoted the binding of EPI with tetracycline and acted on MDR *P. aeruginosa* [143]. They also explored the effect of tobramycin–EPI conjugates in relation to with fluroquinolone, rifampicin, and fosfomycin, showing that they effectively reduced the MIC_80_ of these drugs and exerted a strong joint effect [144]. Adamson et al. showed that the combination of EPI (trimethoprim and sertraline) and levofloxacin against the overexpression of MexAB-OprM pumps in *P. aeruginosa* produced advanced benefit compared with the monotherapy with levofloxacin [145]. Prasch et al. conducted similar research and demonstrated that when the above efflux pump inhibitors are co-administered with antibiotics, the antibiotic therapeutic dose can be reduced [146].

In addition to the EPIs mentioned above, plant-derived EPIs have also been researched (Table 4). Based on various extraction mechanisms, more than 20 different potential plant-derived EPIs have been reported [147,148]. In addition to good anti-inflammatory and antibacterial effects, compounds extracted from vegetal including berberine, Artesunate, and Curcumin inhibit the efflux pump activity of the Gram-negative bacilli *E. coli* and *P. aeruginosa* [149,150,151]. Vegetables (e.g., *Momordica balsamina*), seeds (e.g., milk thistle seeds), spices (e.g., pepper and cumin), essential oils derived from aromatic plants (e.g., trans-cinnamaldehyde and eugenol), etc., are excellent sources of EPIs [147,152,153,154,155,156]. Flavonoids, particularly flavonolignans, were found to have great application prospects in the fight against multidrug resistance by inhibiting bacterial ABC transporters and other efflux pumps [157].

## 5. Conclusions and Perspectives

This work reviewed functional studies of various efflux pump families whose substrates include heavy metals, disinfectants, preservatives, toxins, and virulence factors, in addition to conventional antibiotics. Apart from mediating multidrug resistance, efflux pumps also confer resistance to heavy metals and disinfectants, and even cross-resistance to them. This suggests that efflux pumps have much more complex actions than we initially thought. Efflux pumps play important roles in biological processes such as biofilm formation, quorum sensing, bacterial adhesion to host cells, and invasiveness. This further highlights the importance of developing efflux pump inhibitors. Many discovered efflux pump inhibitors are in clinical trials, though some have even been excluded due to their excessive toxicity. Therefore, there is an urgent need to find safe, green, and harmless efflux pump inhibitors. Plant-derived extracts are interesting candidates. Overall, the functional diversity of efflux pumps remains to be discovered, and the development and utilization of efflux pump inhibitors still require further exploration.

According to preceding research, proteins of the TetR family can be used for the detection of antibiotic residues involved in drug resistance. In addition to high-affinity transcriptional regulatory proteins reported above, there are other potential proteins that recognize a single substrate and have a specific drug binding domain. As shown in Table 3, regulatory and constitutive proteins have specific drug binding sites that can be used for the detection of antibiotics residues involved in the binding. Therefore, efflux pump-related proteins can also be used to detect antibiotic residues, especially transcriptional regulators, though only TetR family proteins are currently applied in practice. It is imperatively demanded that researchers discover more appropriate proteins with high binding specificity for antibiotics. Moreover, this article mentions the connections between the formation of biofilms and the expression of efflux pumps genes, which offers new perspectives to widen fundamental research. This article illustrated the functions of efflux genes and relevant transporters, a topic that requires constant exploration to provide guidance for clinical applications.

## Figures and Tables

**Table 1:** Efflux pumps regulated by the TetR family.

Microorganisms	Efflux Pump	Regulators	Crucial Amino Acid Residues	Substrates	References
*C. jejuni*	CmeABC	CmeR	-	Multidrug	[54]
*E. coli*	AcrB TetA	AcrR TetR	Gly-616 His-64, Thr-103, Arg-104, Pro-105	Macrolide Tetracyclines and Mg^2+^ complex	[65,66]
*L. monocytogenes*	FepA	FepR	-	Fluoroquinolones	[67]
*N. gonorrhoeae*	MtrCDE	MtrR	-	Hydrophobic antibiotics	[59]
*V. cholerae*	NorM	-	Glu-124, Glu190, Asp-155, Gly-184, Gly-187, Lys-185, Pro-189, Cys-196, and Tyr-384	Norfloxacin	[68]
*P. aeruginosa*	MexAB-OprM	MexR	-	Novobiocin	[55,56]
*P. putida*	TtgABC	TtgR	Ser-77, Glu-78, Asn-110, His-114	Tetracyclines, Chloramphenicol	[57]
*S. altophilia*	SmeDEF	SmeT	His-67, Ser-96, His-167	Tetracyclines, Chloramphenicol, Quinolones	[58]

**Table 2:** Efflux pumps present in Gram-negative bacteria.

Efflux Pump Family	Efflux Pump	Regulator	Organisms	Substrates (Class)	Resistance to Specific Antibiotics ^a^	References
ABC	MacAB-TolC	PhoPQ	*E. coli*, *N. gonorrhoeae*, *S. maltophilia*	Macrolides	EM	[18,86]
MATE	NorM	/	*N. gonorrhoeae*	Fluoroquinolones, EB, Rhodamine 6G	NF, CP	[29,33]
MFS	EmrAB-TolC	EmrR	*E. coli*	Cotrimoxazole	/	[40]
	MdfA, MdtM	/		Tigecycline, chloramphenicol	DC, CM	[86,87,88]
	QepA	QepR		Fluoroquinolones	FQ	[89]
	TetA	TetR		Tigecycline	TC	[66]
RND	AcrAB-TolC	AcrR	*E. coli*, *K. pneumoniae*, *S. enterica*	β-lactams, Fluoroquinolones	KF, CM, FQ, P	[90,91]
	AdeABC	AadR, AadS	*A. baumannii*	Aminoglycosides, Erythromycin, Chloramphenicol, Fluoroquinolones, Tetracyclines, Trimethoprim, some β-lactams, Bile salts	AZI	[43]
	CmeABC	CmeR	*C. jejuni*	β-lactams, Tetracyclines, Quinolones	TC	[44]
	MexAB-OprM	NalC/NalD	*P. aeruginosa*	Quinolones	CM, CP, TC, SM	[45]
	MtrCDE	MtrR, MtrR	*N. gonorrhoeae*	Fluoroquinolones	CP, RF	[46]
	OqxAB	OqxR	*E. coli*, *K. pneumoniae*, *S. enterica*	Chloramphenicol, Fluoroquinolones	CM, NT, NF, CP, LEV	[47,48]
	SmeDEF	SmeT	*S. maltophilia*	Aminoglycosides, Trimethoprim Tetracyclines, Chloramphenicol	GM, CZ, IMP, MP, CAR, TC	[49]
	TtgABC	TtgR	*P. putida*	Chloramphenicol	CM, TC	[50]
SMR	AbeS	/	*A. baumannii*	Ethidium, Acriflavine, Benzalkonium	EM, NO	[72,73]
	EmrE	/	*E. coli*, *P. aeruginosa*	Quaternary ammonium compounds	Quaternary ammonium compounds	[75,76]
	KpnEF	/	*K. pneumoniae*	Benzalkonium chloride, Chlorhexidine	CT, EM, RF, TC, SM	[74]

^a^ ACR, acriflavine; ADM, adriamycin; AG, aminoglycosides; AZI, azithromycin; CAR, carbenicillin; CLI, clindamycin; CM, chloramphenicol; CP, ciprofloxacin; CR, clarithromycin; CT, Colistin; CZ, cefoperazone; DA, dalfopristin; DAU, daunomycin; DC, doxycycline; DTM, distamycin; EB, ethidium bromide; EM, erythromycin; FQ, fluoroquinolones; FU, fusidic acid; IMP, imipenem; KF, cephalosporins; GM, gentamicin; LEV, levofloxacin; ML, macrolides; MP, meropenem; NF, norfloxacin; NO, novobiocin; NT, nitrofurantoin; OF, ofloxacin; P, penicillins; RF, rifampicin; ROX, roxithromycin; SM, streptomycin; TC, tetracycline; TM, trimethoprim. “/” mean no transcription regulators found.

**Table 3:** Efflux pumps present in Gram-positive bacteria.

Efflux Pump Family	Efflux Pump	Regulator	Organisms	Substrates	Resistance to Specific Antibiotics ^a^	Reference
ABC	EfrAB	/	*E. faecalis*	acriflavine, ethidium bromide, safranin O, DAPI, daunomycin, doxorubicin, novobiocin, arbekacin, doxycycline and norfloxacin	GM, SM, CM	[24]
	LmrA	/	*L. lactis*	Macrolides, Lincosamides, Streptogramins	DAU, ADM	[21,25]
	Msr	/	*Streptococcus*	Macrolides	ML	[22,26,27]
	PatA/PatB	/	*S. pneumoniaee*	Fluoroquinolones	FQ	[23,28]
MATE	FepA	FepR	*L. monocytogenes*	Fluoroquinolones	NF, CP	[64]
	MepA	MepR	*S. aureus*	Fluoroquinolones, Tigecycline, Pentamidine	DT	[92,93]
MFS	Lde	/	*L. monocytogenes*	Fluoroquinolones	ACR, EB	[36]
	NorA, NorB, NorC	MgrA, NorG, ArlRS	*S. aureus*	Fluoroquinolones	NF, CP	[37]
	Mef	/	*S. pneumoniae*	Macrolides	EM, AZI, ROX, CR	[38,39,40]
SMR	Qac	QacR	*S. aureus*, *Enterococcus* spp., *E. faecalis*	Quaternary ammonium compounds	Quaternary ammonium compounds	[77]

^a^ ACR, acriflavine; ADM, adriamycin; AZI, azithromycin; CP, ciprofloxacin; CR, clarithromycin; CM, chloramphenicol; DA, dalfopristin; DAU, daunomycin; DT, dirithromycin; EB, ethidium bromide; EM, erythromycin; GM, gentamicin; FQ, fluoroquinolones; ML, macrolides; NF, norfloxacin; ROX, roxithromycin; SM, streptomycin.

**Table 4:** Plant-derived EPIs.

Bioactive Compounds	Bacterial Species	Pharmacological Activity	References
Berberine	*P. aeruginosa*	Inhibited the multidrug efflux system MexXY-OprM	[149]
Artesunate	*E. coli*	Inhibited the multidrug efflux pump system AcrAB-TolC	[151]
Curcumin	*P. aeruginosa*	Inhibited the expression of efflux pump	[150]
plant-derived flavonoids such as skullcapflavone II and nobiletin	*Mycobacterial Species*	Inhibited the activity of the efflux pump and decreased the rifampicin-resistance level	[153]
Extracts of milk thistle seeds and reserpine	*Salmonella Typhi*	Inhibited an efflux transporter STY4874	[154]
*Hypericum olympicum* L. cf. uniflorum-derived natural product	*S. aureus*	Inhibited NorA multidrug efflux pump activity	[147]
diterpene isolated from Chamaecyparis lawsoniana: ferruginol	Methicillin-resistant *S. aureus* (MRSA)	Inhibited the TetK pump	[155]
quinine isolated from Cinchona tree’s bark		Inhibited the activity of the efflux pump	
piperine isolated from the Piperaceae family			
harmaline isolated from Perganum harmala			
4′,5′-O-dicaffeoylquinic acid isolated from wormwood (Artemisia absinthium)			
triterpenoids from Momordica balsamina			
carnosic acid from Rosmarinus officinalis			
coumarins from Mesua ferrea			
clerodane diterpene from Polyalthia longifolia		Downregulation of MFS and MATE family efflux genes such as *norA*, *norB*, *norC*, *mdeA*, and *mepA*	
cumin spice (Cuminum cyminum)		inhibited LmrS drug transport (a proton-driven multidrug efflux pump in MRSA)	
trans-cinnamaldehyde and eugenol	*A. baumannii*	downregulated the expression of efflux pump-related gene *adeABC*	

## Data Availability

Not applicable.

## Abbreviations

ABC	ATP-binding cassette;
ATP	adenosine triphosphate;
EPI	efflux pump inhibitor;
HTH	helix–turn–helix;
IMF	inner-membrane fusion protein;
MATE	multidrug and toxic microbial extrusion;
MDR	multidrug resistant;
MFP	membrane fusion protein;
MFS	major facilitator super family;
Mg	magnesium;
MIC	minimum inhibitory concentration;
OMF	outer membrane protein;
PACE	Proteobacterial Antimicrobial Compound Efflux;
QS	Quorum sensing;
RND	resistance nodulation and cell division;
SMR	small multidrug resistance;
SNP	single-nucleotide polymorphism.

## References

[1] Ganas P., Mihasan M., Igloi G.L., Brandsch R. (2007). A two-component small multidrug resistance pump functions as a metabolic valve during nicotine catabolism by *Arthrobacter nicotinovorans*. Microbiology.

[2] Hernando-Amado S., Blanco P., Alcalde-Rico M., Corona F., Reales-Calderon J.A., Sanchez M.B., Martinez J.L. (2016). Multidrug efflux pumps as main players in intrinsic and acquired resistance to antimicrobials. Drug Resist. Updat..

[3] Blair J.M., Piddock L.J. (2016). How to Measure Export via Bacterial Multidrug Resistance Efflux Pumps. mBio.

[4] Kong E.F., Tsui C., Kucharikova S., Van Dijck P., Jabra-Rizk M.A. (2017). Modulation of *Staphylococcus aureus* Response to Antimicrobials by the Candida albicans Quorum Sensing Molecule Farnesol. Antimicrob. Agents Chemother..

[5] Alcalde-Rico M., Hernando-Amado S., Blanco P., Martinez J.L. (2016). Multidrug Efflux Pumps at the Crossroad between Antibiotic Resistance and Bacterial Virulence. Front. Microbiol..

[6] Piddock L.J. (2006). Multidrug-resistance efflux pumps—Not just for resistance. Nat. Rev. Microbiol..

[7] Hassan K.A., Liu Q., Elbourne L.D.H., Ahmad I., Sharples D., Naidu V., Chan C.L., Li L., Harborne S.P.D., Pokhrel A. (2018). Pacing across the membrane: The novel PACE family of efflux pumps is widespread in Gram-negative pathogens. Res. Microbiol..

[8] Li L., Tetu S.G., Paulsen I.T., Hassan K.A. (2018). A Transcriptomic Approach to Identify Novel Drug Efflux Pumps in Bacteria. Methods Mol. Biol..

[9] Verchere A., Broutin I., Picard M. (2012). Photo-induced proton gradients for the in vitro investigation of bacterial efflux pumps. Sci. Rep..

[10] Kim Y.C., Hummer G. (2012). Proton-pumping mechanism of cytochrome c oxidase: A kinetic master-equation approach. Biochim. Biophys. Acta.

[11] Daury L., Orange F., Taveau J.C., Verchere A., Monlezun L., Gounou C., Marreddy R.K., Picard M., Broutin I., Pos K.M. (2016). Tripartite assembly of RND multidrug efflux pumps. Nat. Commun..

[12] Neuberger A., Du D., Luisi B.F. (2018). Structure and mechanism of bacterial tripartite efflux pumps. Res. Microbiol..

[13] Krishnamoorthy G., Weeks J.W., Zhang Z., Chandler C.E., Xue H., Schweizer H.P., Ernst R.K., Zgurskaya H.I. (2019). Efflux Pumps of *Burkholderia thailandensis* Control the Permeability Barrier of the Outer Membrane. Antimicrob. Agents Chemother..

[14] Kumar S., Lekshmi M., Parvathi A., Ojha M., Wenzel N., Varela M.F. (2020). Functional and Structural Roles of the Major Facilitator Superfamily Bacterial Multidrug Efflux Pumps. Microorganisms.

[15] Colclough A.L., Alav I., Whittle E.E., Pugh H.L., Darby E.M., Legood S.W., McNeil H.E., Blair J.M. (2020). RND efflux pumps in Gram-negative bacteria; regulation, structure and role in antibiotic resistance. Future Microbiol..

[16] Jo I., Hong S., Lee M., Song S., Kim J.S., Mitra A.K., Hyun J., Lee K., Ha N.C. (2017). Stoichiometry and mechanistic implications of the MacAB-TolC tripartite efflux pump. Biochem. Biophys. Res. Commun..

[17] Fitzpatrick A.W.P., Llabrés S., Neuberger A., Blaza J.N., Bai X.C., Okada U., Murakami S., van Veen H.W., Zachariae U., Scheres S.H.W. (2017). Structure of the MacAB-TolC ABC-type tripartite multidrug efflux pump. Nat. Microbiol..

[18] Lu S., Zgurskaya H.I. (2013). MacA, a periplasmic membrane fusion protein of the macrolide transporter MacAB-TolC, binds lipopolysaccharide core specifically and with high affinity. J. Bacteriol..

[19] Shirshikova T.V., Sierra-Bakhshi C.G., Kamaletdinova L.K., Matrosova L.E., Khabipova N.N., Evtugyn V.G., Khilyas I.V., Danilova I.V., Mardanova A.M., Sharipova M.R. (2021). The ABC-Type Efflux Pump MacAB Is Involved in Protection of *Serratia marcescens* against Aminoglycoside Antibiotics, Polymyxins, and Oxidative Stress. mSphere.

[20] Shi K., Cao M., Li C., Huang J., Zheng S., Wang G. (2019). Efflux proteins MacAB confer resistance to arsenite and penicillin/macrolide-type antibiotics in *Agrobacterium tumefaciens* 5A. World J. Microbiol. Biotechnol..

[21] Hellmich U.A., Monkemeyer L., Velamakanni S., van Veen H.W., Glaubitz C. (2015). Effects of nucleotide binding to LmrA: A combined MAS-NMR and solution NMR study. Biochim. Biophys. Acta.

[22] Iannelli F., Santagati M., Santoro F., Oggioni M.R., Stefani S., Pozzi G. (2014). Nucleotide sequence of conjugative prophage Phi1207.3 (formerly Tn1207.3) carrying the mef(A)/msr(D) genes for efflux resistance to macrolides in *Streptococcus pyogenes*. Front. Microbiol..

[23] Garvey M.I., Baylay A.J., Wong R.L., Piddock L.J. (2011). Overexpression of *patA* and *patB*, which encode ABC transporters, is associated with fluoroquinolone resistance in clinical isolates of *Streptococcus pneumoniae*. Antimicrob. Agents Chemother..

[24] Lavilla Lerma L., Benomar N., Valenzuela A.S., Casado Munoz Mdel C., Galvez A., Abriouel H. (2014). Role of EfrAB efflux pump in biocide tolerance and antibiotic resistance of *Enterococcus faecalis* and *Enterococcus faecium* isolated from traditional fermented foods and the effect of EDTA as EfrAB inhibitor. Food Microbiol..

[25] Hellmich U.A., Lyubenova S., Kaltenborn E., Doshi R., van Veen H.W., Prisner T.F., Glaubitz C. (2012). Probing the ATP hydrolysis cycle of the ABC multidrug transporter LmrA by pulsed EPR spectroscopy. J. Am. Chem. Soc..

[26] Zhang Y., Tatsuno I., Okada R., Hata N., Matsumoto M., Isaka M., Isobe K., Hasegawa T. (2016). Predominant role of *msr(D)* over *mef(A)* in macrolide resistance in *Streptococcus pyogenes*. Microbiology.

[27] Tatsuno I., Isaka M., Masuno K., Hata N., Matsumoto M., Hasegawa T. (2018). Functional Predominance of *msr(D)*, Which Is More Effective as mef(A)-Associated Than mef(E)-Associated, Over mef(A)/mef(E) in Macrolide Resistance in *Streptococcus pyogenes*. Microb. Drug Resist..

[28] Baylay A.J., Piddock L.J. (2015). Clinically relevant fluoroquinolone resistance due to constitutive overexpression of the PatAB ABC transporter in *Streptococcus pneumoniae* is conferred by disruption of a transcriptional attenuator. J. Antimicrob. Chemother..

[29] Du D., van Veen H.W., Murakami S., Pos K.M., Luisi B.F. (2015). Structure, mechanism and cooperation of bacterial multidrug transporters. Curr. Opin. Struct. Biol..

[30] Lu M. (2016). Structures of multidrug and toxic compound extrusion transporters and their mechanistic implications. Channels.

[31] Kuroda T., Tsuchiya T. (2009). Multidrug efflux transporters in the MATE family. Biochim. Biophys. Acta.

[32] Kusakizako T., Miyauchi H., Ishitani R., Nureki O. (2020). Structural biology of the multidrug and toxic compound extrusion superfamily transporters. Biochim. Biophys. Acta Biomembr..

[33] Rouquette-Loughlin C.E., Dhulipala V., Reimche J.L., Raterman E., Begum A.A., Jerse A.E., Shafer W.M. (2018). cis- and trans-Acting Factors Influence Expression of the norM-Encoded Efflux Pump of *Neisseria gonorrhoeae* and Levels of *Gonococcal* Susceptibility to Substrate Antimicrobials. Antimicrob. Agents Chemother..

[34] Guelfo J.R., Rodríguez-Rojas A., Matic I., Blázquez J. (2010). A MATE-family efflux pump rescues the *Escherichia coli* 8-oxoguanine-repair-deficient mutator phenotype and protects against H(2)O(2) killing. PLoS Genet..

[35] Tocci N., Iannelli F., Bidossi A., Ciusa M.L., Decorosi F., Viti C., Pozzi G., Ricci S., Oggioni M.R. (2013). Functional analysis of pneumococcal drug efflux pumps associates the MATE DinF transporter with quinolone susceptibility. Antimicrob. Agents Chemother..

[36] Jiang X., Zhou L., Gao D., Wang Y., Wang D., Zhang Z., Chen M., Su Y., Li L., Yan H. (2012). Expression of efflux pump gene lde in ciprofloxacin-resistant foodborne isolates of *Listeria monocytogenes*. Microbiol. Immunol..

[37] Costa S.S., Viveiros M., Amaral L., Couto I. (2013). Multidrug Efflux Pumps in *Staphylococcus aureus*: An Update. Open Microbiol. J..

[38] Bley C., van der Linden M., Reinert R.R. (2011). mef(A) is the predominant macrolide resistance determinant in *Streptococcus pneumoniae* and *Streptococcus pyogenes* in Germany. Int. J. Antimicrob. Agents.

[39] Chancey S.T., Bai X.H., Kumar N., Drabek E.F., Daugherty S.C., Colon T., Ott S., Sengamalay N., Sadzewicz L., Tallon L.J. (2015). Transcriptional Attenuation Controls Macrolide Inducible Efflux and Resistance in *Streptococcus pneumoniae* and in Other Gram-Positive Bacteria Containing mef/mel (msr(D)) Elements. PLoS ONE.

[40] Nunez-Samudio V., Chesneau O. (2013). Functional interplay between the ATP binding cassette Msr(D) protein and the membrane facilitator superfamily Mef(E) transporter for macrolide resistance in *Escherichia coli*. Res. Microbiol..

[41] Pasqua M., Bonaccorsi di Patti M.C., Fanelli G., Utsumi R., Eguchi Y., Trirocco R., Prosseda G., Grossi M., Colonna B. (2021). Host-Bacterial Pathogen Communication: The Wily Role of the Multidrug Efflux Pumps of the MFS Family. Front. Mol. Biosci..

[42] Pérez-Varela M., Corral J., Aranda J., Barbé J. (2019). Roles of Efflux Pumps from Different Superfamilies in the Surface-Associated Motility and Virulence of *Acinetobacter baumannii* ATCC 17978. Antimicrob. Agents Chemother..

[43] Leus I.V., Weeks J.W., Bonifay V., Smith L., Richardson S., Zgurskaya H.I. (2018). Substrate specificities and efflux efficiencies of RND efflux pumps of *Acinetobacter baumannii*. J. Bacteriol..

[44] Perez-Boto D., Acebo P., Garcia-Pena F.J., Abad J.C., Echeita M.A., Amblar M. (2015). Isolation of a point mutation associated with altered expression of the CmeABC efflux pump in a multidrug-resistant *Campylobacter jejuni* population of poultry origin. J. Glob. Antimicrob. Resist..

[45] Dreier J., Ruggerone P. (2015). Interaction of antibacterial compounds with RND efflux pumps in *Pseudomonas aeruginosa*. Front. Microbiol..

[46] Castanheira M., Deshpande L.M., Jones R.N., Farrell D.J. (2012). Evaluation of quinolone resistance-determining region mutations and efflux pump expression in *Neisseria meningitidis* resistant to fluoroquinolones. Diagn. Microbiol. Infect. Dis..

[47] Yuan J., Xu X., Guo Q., Zhao X., Ye X., Guo Y., Wang M. (2012). Prevalence of the oqxAB gene complex in *Klebsiella pneumoniae* and *Escherichia coli* clinical isolates. J. Antimicrob. Chemother..

[48] Wong M.H., Chan E.W., Xie L., Li R., Chen S. (2016). IncHI2 Plasmids Are the Key Vectors Responsible for oqxAB Transmission among *Salmonella* Species. Antimicrob. Agents Chemother..

[49] Cho H.H., Sung J.Y., Kwon K.C., Koo S.H. (2012). Expression of Sme efflux pumps and multilocus sequence typing in clinical isolates of *Stenotrophomonas maltophilia*. Ann. Lab. Med..

[50] Basler G., Thompson M., Tullman-Ercek D., Keasling J. (2018). A *Pseudomonas putida* efflux pump acts on short-chain alcohols. Biotechnol. Biofuels.

[51] Verma P., Maurya P., Tiwari M., Tiwari V. (2017). In-silico interaction studies suggest RND efflux pump mediates polymyxin resistance in *Acinetobacter baumannii*. J. Biomol. Struct. Dyn..

[52] Blair J.M., Bavro V.N., Ricci V., Modi N., Cacciotto P., Kleinekathfer U., Ruggerone P., Vargiu A.V., Baylay A.J., Smith H.E. (2015). AcrB drug-binding pocket substitution confers clinically relevant resistance and altered substrate specificity. Proc. Natl. Acad. Sci. USA.

[53] Manjasetty B.A., Halavaty A.S., Luan C.H., Osipiuk J., Mulligan R., Kwon K., Anderson W.F., Joachimiak A. (2016). Loop-to-helix transition in the structure of multidrug regulator AcrR at the entrance of the drug-binding cavity. J. Struct. Biol..

[54] Routh M.D., Su C.C., Zhang Q., Yu E.W. (2009). Structures of AcrR and CmeR: Insight into the mechanisms of transcriptional repression and multi-drug recognition in the TetR family of regulators. Biochim. Biophys. Acta.

[55] Ghosh S., Cremers C.M., Jakob U., Love N.G. (2011). Chlorinated phenols control the expression of the multidrug resistance efflux pump MexAB-OprM in *Pseudomonas aeruginosa* by interacting with NalC. Mol. Microbiol..

[56] Chen W., Wang D., Zhou W., Sang H., Liu X., Ge Z., Zhang J., Lan L., Yang C.G., Chen H. (2016). Novobiocin binding to NalD induces the expression of the MexAB-OprM pump in *Pseudomonas aeruginosa*. Mol. Microbiol..

[57] Fernandez-Escamilla A.M., Fernandez-Ballester G., Morel B., Casares-Atienza S., Ramos J.L. (2015). Molecular Binding Mechanism of TtgR Repressor to Antibiotics and Antimicrobials. PLoS ONE.

[58] Hernandez A., Mate M.J., Sanchez-Diaz P.C., Romero A., Rojo F., Martinez J.L. (2009). Structural and functional analysis of SmeT, the repressor of the *Stenotrophomonas maltophilia* multidrug efflux pump SmeDEF. J. Biol. Chem..

[59] Johnson P.J., Shafer W.M. (2015). The Transcriptional Repressor, MtrR, of the mtrCDE Efflux Pump Operon of *Neisseria gonorrhoeae* Can Also Serve as an Activator of “off Target” Gene (glnE) Expression. Antibiotics.

[60] Anandapadamanaban M., Pilstal R., Andresen C., Trewhella J., Moche M., Wallner B., Sunnerhagen M. (2016). Mutation-Induced Population Shift in the MexR Conformational Ensemble Disengages DNA Binding: A Novel Mechanism for MarR Family Derepression. Structure.

[61] Xu S., Chen G., Liu Z., Xu D., Wu Z., Li Z., Hong M. (2020). Site-Directed Mutagenesis Reveals Crucial Residues in *Escherichia coli* Resistance-Nodulation-Division Efflux Pump OqxB. Microb. Drug Resist..

[62] Lv Z., Zhao M., Wang W., Wang Q., Huang M., Li C., Lian Q., Xia J., Qi J., Xiang C. (2021). Changing Gly311 to an acidic amino acid in the MATE family protein DTX6 enhances Arabidopsis resistance to the dihydropyridine herbicides. Mol. Plant.

[63] Bhakdi S., Mackman N., Menestrina G., Gray L., Hugo F., Seeger W., Holland I.B. (1988). The hemolysin of *Escherichia coli*. Eur. J. Epidemiol..

[64] Gilson L., Mahanty H.K., Kolter R. (1990). Genetic analysis of an MDR-like export system: The secretion of colicin V. EMBO J..

[65] Wehmeier C., Schuster S., Fähnrich E., Kern W.V., Bohnert J.A. (2009). Site-directed mutagenesis reveals amino acid residues in the *Escherichia coli* RND efflux pump AcrB that confer macrolide resistance. Antimicrob. Agents Chemother..

[66] Wright D.J., Tate C.G. (2015). Isolation and characterisation of transport-defective substrate-binding mutants of the tetracycline antiporter TetA(B). Biochim. Biophys. Acta.

[67] Guérin F., Galimand M., Tuambilangana F., Courvalin P., Cattoir V. (2014). Overexpression of the novel MATE fluoroquinolone efflux pump FepA in *Listeria monocytogenes* is driven by inactivation of its local repressor FepR. PLoS ONE.

[68] Singh A.K., Haldar R., Mandal D., Kundu M. (2006). Analysis of the topology of *Vibrio cholerae* NorM and identification of amino acid residues involved in norfloxacin resistance. Antimicrob. Agents Chemother..

[69] Delmar J.A., Su C.C., Yu E.W. (2014). Bacterial multidrug efflux transporters. Annu. Rev. Biophys..

[70] Long F., Su C.C., Lei H.T., Bolla J.R., Do S.V., Yu E.W. (2012). Structure and mechanism of the tripartite CusCBA heavy-metal efflux complex. Philos. Trans. R. Soc. Lond. Ser. B Biol. Sci..

[71] Blanco P., Hernando-Amado S., Reales-Calderon J.A., Corona F., Lira F., Alcalde-Rico M., Bernardini A., Sanchez M.B., Martinez J.L. (2016). Bacterial Multidrug Efflux Pumps: Much More Than Antibiotic Resistance Determinants. Microorganisms.

[72] Zwama M., Nishino K. (2021). Ever-Adapting RND Efflux Pumps in Gram-Negative Multidrug-Resistant Pathogens: A Race against Time. Antibiotics.

[73] Bay D.C., Rommens K.L., Turner R.J. (2008). Small multidrug resistance proteins: A multidrug transporter family that continues to grow. Biochim. Biophys. Acta.

[74] Bay D.C., Turner R.J. (2009). Diversity and evolution of the small multidrug resistance protein family. BMC Evol. Biol..

[75] Lytvynenko I., Brill S., Oswald C., Pos K.M. (2016). Molecular basis of polyspecificity of the Small Multidrug Resistance Efflux Pump AbeS from *Acinetobacter baumannii*. J. Mol. Biol..

[76] Lin M.F., Lin Y.Y., Tu C.C., Lan C.Y. (2017). Distribution of different efflux pump genes in clinical isolates of multidrug-resistant *Acinetobacter baumannii* and their correlation with antimicrobial resistance. J. Microbiol. Immunol. Infect. = Wei Mian Yu Gan Ran Za Zhi.

[77] Srinivasan V.B., Rajamohan G. (2013). KpnEF, a new member of the Klebsiella pneumoniae cell envelope stress response regulon, is an SMR-type efflux pump involved in broad-spectrum antimicrobial resistance. Antimicrob. Agents Chemother..

[78] Banigan J.R., Gayen A., Cho M.K., Traaseth N.J. (2015). A structured loop modulates coupling between the substrate-binding and dimerization domains in the multidrug resistance transporter EmrE. J. Biol. Chem..

[79] Padariya M., Kalathiya U., Baginski M. (2017). Structural and dynamic insights on the EmrE protein with TPP(+) and related substrates through molecular dynamics simulations. Chem. Phys. Lipids.

[80] Jaglic Z., Cervinkova D. (2012). Genetic basis of resistance to quaternary ammonium compounds—The qac genes and their role: A review. Vet. Med..

[81] Buffet-Bataillon S., Tattevin P., Maillard J.Y., Bonnaure-Mallet M., Jolivet-Gougeon A. (2016). Efflux pump induction by quaternary ammonium compounds and fluoroquinolone resistance in bacteria. Future Microbiol..

[82] Bolla J.R., Howes A.C., Fiorentino F., Robinson C.V. (2020). Assembly and regulation of the chlorhexidine-specific efflux pump AceI. Proc. Natl. Acad. Sci. USA.

[83] Hassan K.A., Elbourne L.D., Li L., Gamage H.K., Liu Q., Jackson S.M., Sharples D., Kolsto A.B., Henderson P.J., Paulsen I.T. (2015). An ace up their sleeve: A transcriptomic approach exposes the AceI efflux protein of *Acinetobacter baumannii* and reveals the drug efflux potential hidden in many microbial pathogens. Front. Microbiol..

[84] Coenye T., Van Acker H., Peeters E., Sass A., Buroni S., Riccardi G., Mahenthiralingam E. (2011). Molecular mechanisms of chlorhexidine tolerance in *Burkholderia cenocepacia* biofilms. Antimicrob. Agents Chemother..

[85] Nde C.W., Jang H.J., Toghrol F., Bentley W.E. (2009). Global transcriptomic response of *Pseudomonas aeruginosa* to chlorhexidine diacetate. Environ. Sci. Technol..

[86] Lin H.T., Bavro V.N., Barrera N.P., Frankish H.M., Velamakanni S., van Veen H.W., Robinson C.V., Borges-Walmsley M.I., Walmsley A.R. (2009). MacB ABC transporter is a dimer whose ATPase activity and macrolide-binding capacity are regulated by the membrane fusion protein MacA. J. Biol. Chem..

[87] Fluman N., Adler J., Rotenberg S.A., Brown M.H., Bibi E. (2014). Export of a single drug molecule in two transport cycles by a multidrug efflux pump. Nat. Commun..

[88] Fluman N., Bibi E. (2009). Bacterial multidrug transport through the lens of the major facilitator superfamily. Biochim. Biophys. Acta.

[89] Rincón G., Radice M., Giovanakis M., Di Conza J.A., Gutkind G. (2014). First report of plasmid-mediated fluoroquinolone efflux pump QepA in *Escherichia coli* clinical isolate ST68, in South America. Diagn. Microbiol. Infect. Dis..

[90] Atac N., Kurt-Azap O., Dolapci I., Yesilkaya A., Ergonul O., Gonen M., Can F. (2018). The Role of AcrAB-TolC Efflux Pumps on Quinolone Resistance of *E. coli* ST131. Curr. Microbiol..

[91] Saw H.T., Webber M.A., Mushtaq S., Woodford N., Piddock L.J. (2016). Inactivation or inhibition of AcrAB-TolC increases resistance of carbapenemase-producing *Enterobacteriaceae* to carbapenems. J. Antimicrob. Chemother..

[92] Dabul A.N.G., Avaca-Crusca J.S., Van Tyne D., Gilmore M.S., Camargo I. (2018). Resistance in In Vitro Selected Tigecycline-Resistant Methicillin-Resistant *Staphylococcus aureus* Sequence Type 5 Is Driven by Mutations in mepR and mepA Genes. Microb. Drug Resist..

[93] Banchs C., Poulos S., Nimjareansuk W.S., Joo Y.E., Faham S. (2014). Substrate binding to the multidrug transporter MepA. Biochim. Biophys. Acta.

[94] Hall C.W., Mah T.F. (2017). Molecular mechanisms of biofilm-based antibiotic resistance and tolerance in pathogenic bacteria. FEMS Microbiol. Rev..

[95] Alav I., Sutton J.M., Rahman K.M. (2018). Role of bacterial efflux pumps in biofilm formation. J. Antimicrob. Chemother..

[96] Chen H.L., Cao J.M., Zhou C., Liu H.Y., Zhang X.X., Zhou T.L. (2017). Biofilm Formation Restrained by Subinhibitory Concentrations of Tigecyclin in *Acinetobacter baumannii* Is Associated with Downregulation of Efflux Pumps. Chemotherapy.

[97] Gupta D., Singh A., Khan A.U. (2017). Nanoparticles as Efflux Pump and Biofilm Inhibitor to Rejuvenate Bactericidal Effect of Conventional Antibiotics. Nanoscale Res. Lett..

[98] Sabatini S., Piccioni M., Felicetti T., De Marco S., Manfroni G., Pagiotti R., Nocchetti M., Cecchetti V., Pietrella D. (2017). Investigation on the effect of known potent *S. aureus* NorA efflux pump inhibitors on the *staphylococcal* biofilm formation. RSC Adv..

[99] Robin B., Nicol M., Le H., Tahrioui A., Schaumann A., Vuillemenot J.B., Vergoz D., Lesouhaitier O., Jouenne T., Hardouin J. (2021). MacAB-TolC Contributes to the Development of *Acinetobacter baumannii* Biofilm at the Solid-Liquid Interface. Front. Microbiol..

[100] Yoon E.J., Balloy V., Fiette L., Chignard M., Courvalin P., Grillot-Courvalin C. (2016). Contribution of the Ade Resistance-Nodulation-Cell Division-Type Efflux Pumps to Fitness and Pathogenesis of *Acinetobacter baumannii*. mBio.

[101] Dou Y., Song F., Guo F., Zhou Z.D., Zhu C.L., Xiang J., Huan J.N. (2017). *Acinetobacter baumannii* quorum-sensing signalling molecule induces the expression of drug-resistance genes. Mol. Med. Rep..

[102] Richmond G.E., Evans L.P., Anderson M.J., Wand M.E., Bonney L.C., Ivens A., Chua K.L., Webber M.A., Sutton J.M., Peterson M.L. (2016). The *Acinetobacter baumannii* Two-Component System AdeRS Regulates Genes Required for Multidrug Efflux, Biofilm Formation, and Virulence in a Strain-Specific Manner. mBio.

[103] Sato Y., Unno Y., Ubagai T., Ono Y. (2018). Sub-minimum inhibitory concentrations of colistin and polymyxin B promote *Acinetobacter baumannii* biofilm formation. PLoS ONE.

[104] Sakhtah H., Koyama L., Zhang Y., Morales D.K., Fields B.L., Price-Whelan A., Hogan D.A., Shepard K., Dietrich L.E.P. (2016). The *Pseudomonas aeruginosa* efflux pump MexGHI-OpmD transports a natural phenazine that controls gene expression and biofilm development. Proc. Natl. Acad. Sci. USA.

[105] Liang Z.B., Chen Y.M., Chen Y., Cheng Y.Y., Zhang L.H. (2016). RND efflux pump and its interrelationship with quorum sensing system. Yi Chuan = Hered..

[106] Wolloscheck D., Krishnamoorthy G., Nguyen J., Zgurskaya H.I. (2018). Kinetic Control of Quorum Sensing in *Pseudomonas aeruginosa* by Multidrug Efflux Pumps. ACS Infect. Dis..

[107] Cannon R.D., Holmes A.R. (2015). Learning the ABC of oral fungal drug resistance. Mol. Oral. Microbiol..

[108] Santiago A.E., Yan M.B., Tran M., Wright N., Luzader D.H., Kendall M.M., Ruiz-Perez F., Nataro J.P. (2016). A large family of anti-activators accompanying XylS/AraC family regulatory proteins. Mol. Microbiol..

[109] Deochand D.K., Grove A. (2017). MarR family transcription factors: Dynamic variations on a common scaffold. Crit. Rev. Biochem. Mol. Biol..

[110] Chang C.C., Lin L.Y., Zou X.W., Huang C.C., Chan N.L. (2015). Structural basis of the mercury(II)-mediated conformational switching of the dual-function transcriptional regulator MerR. Nucleic Acids Res..

[111] Deng W., Li C., Xie J. (2013). The underling mechanism of bacterial TetR/AcrR family transcriptional repressors. Cell Signal.

[112] Hwang S., Zhang Q., Ryu S., Jeon B. (2012). Transcriptional regulation of the CmeABC multidrug efflux pump and the KatA catalase by CosR in *Campylobacter jejuni*. J. Bacteriol..

[113] Juarez P., Jeannot K., Plesiat P., Llanes C. (2017). Toxic Electrophiles Induce Expression of the Multidrug Efflux Pump MexEF-OprN in Pseudomonas aeruginosa through a Novel Transcriptional Regulator, CmrA. Antimicrob. Agents Chemother..

[114] Hernandez A., Ruiz F.M., Romero A., Martinez J.L. (2011). The binding of triclosan to SmeT, the repressor of the multidrug efflux pump SmeDEF, induces antibiotic resistance in *Stenotrophomonas maltophilia*. PLoS Pathog..

[115] Ruiz C., Levy S.B. (2014). Regulation of acrAB expression by cellular metabolites in *Escherichia coli*. J. Antimicrob. Chemother..

[116] Sun J.R., Chiang Y.S., Shang H.S., Perng C.L., Yang Y.S., Chiueh T.S. (2017). Phenotype microarray analysis of the AdeRS two-component system in *Acinetobacter baumannii*. Eur. J. Clin. Microbiol. Infect. Dis..

[117] Lin M.F., Lin Y.Y., Lan C.Y. (2015). The Role of the Two-Component System BaeSR in Disposing Chemicals through Regulating Transporter Systems in *Acinetobacter baumannii*. PLoS ONE.

[118] Wu C.J., Huang Y.W., Lin Y.T., Ning H.C., Yang T.C. (2016). Inactivation of SmeSyRy Two-Component Regulatory System Inversely Regulates the Expression of SmeYZ and SmeDEF Efflux Pumps in *Stenotrophomonas maltophilia*. PLoS ONE.

[119] Lin Y.T., Huang Y.W., Liou R.S., Chang Y.C., Yang T.C. (2014). MacABCsm, an ABC-type tripartite efflux pump of *Stenotrophomonas maltophilia* involved in drug resistance, oxidative and envelope stress tolerances and biofilm formation. J. Antimicrob. Chemother..

[120] Walker J.N., Crosby H.A., Spaulding A.R., Salgado-Pabon W., Malone C.L., Rosenthal C.B., Schlievert P.M., Boyd J.M., Horswill A.R. (2013). The *Staphylococcus aureus* ArlRS two-component system is a novel regulator of agglutination and pathogenesis. PLoS Pathog..

[121] Nishino K., Yamasaki S., Nakashima R., Zwama M., Hayashi-Nishino M. (2021). Function and Inhibitory Mechanisms of Multidrug Efflux Pumps. Front. Microbiol..

[122] Nishino K. (2018). Regulation of the Expression of Bacterial Multidrug Exporters by Two-Component Signal Transduction Systems. Methods Mol. Biol..

[123] Henderson P.J.F., Maher C., Elbourne L.D.H., Eijkelkamp B.A., Paulsen I.T., Hassan K.A. (2021). Physiological Functions of Bacterial “Multidrug” Efflux Pumps. Chem. Rev..

[124] Li X.Z., Plesiat P., Nikaido H. (2015). The challenge of efflux-mediated antibiotic resistance in Gram-negative bacteria. Clin. Microbiol. Rev..

[125] Goli H.R., Nahaei M.R., Rezaee M.A., Hasani A., Samadi Kafil H., Aghazadeh M., Sheikhalizadeh V. (2016). Contribution of mexAB-oprM and mexXY (-oprA) efflux operons in antibiotic resistance of clinical *Pseudomonas aeruginosa* isolates in Tabriz, Iran. Infect. Genet. Evol. J. Mol. Epidemiol. Evol. Genet. Infect. Dis..

[126] Murugan N., Malathi J., Therese K.L., Madhavan H.N. (2018). Application of six multiplex PCR’s among 200 clinical isolates of *Pseudomonas aeruginosa* for the detection of 20 drug resistance encoding genes. Kaohsiung J. Med. Sci..

[127] Zhang Y., Li Z., He X., Ding F., Wu W., Luo Y., Fan B., Cao H. (2017). Overproduction of efflux pumps caused reduced susceptibility to carbapenem under consecutive imipenem-selected stress in *Acinetobacter baumannii*. Infect. Drug Resist..

[128] Gerson S., Nowak J., Zander E., Ertel J., Wen Y., Krut O., Seifert H., Higgins P.G. (2018). Diversity of mutations in regulatory genes of resistance-nodulation-cell division efflux pumps in association with tigecycline resistance in *Acinetobacter baumannii*. J. Antimicrob. Chemother..

[129] Nagano K., Nikaido H. (2009). Kinetic behavior of the major multidrug efflux pump AcrB of *Escherichia coli*. Proc. Natl. Acad. Sci. USA.

[130] Wang J., Guo Z.W., Zhi C.P., Yang T., Zhao J.J., Chen X.J., Zeng L., Lv L.C., Zeng Z.L., Liu J.H. (2017). Impact of plasmid-borne oqxAB on the development of fluoroquinolone resistance and bacterial fitness in *Escherichia coli*. J. Antimicrob. Chemother..

[131] Huang J.J., Hu H.Y., Wu Y.H., Wei B., Lu Y. (2013). Effect of chlorination and ultraviolet disinfection on tetA-mediated tetracycline resistance of *Escherichia coli*. Chemosphere.

[132] Melamed S., Naftaly S., Belkin S. (2014). Improved detection of antibiotic compounds by bacterial reporter strains achieved by manipulations of membrane permeability and efflux capacity. Appl. Microbiol. Biotechnol..

[133] Hong H., Park W. (2014). TetR repressor-based bioreporters for the detection of doxycycline using *Escherichia coli* and *Acinetobacter oleivorans*. Appl. Microbiol. Biotechnol..

[134] Weber C.C., Link N., Fux C., Zisch A.H., Weber W., Fussenegger M. (2005). Broad-spectrum protein biosensors for class-specific detection of antibiotics. Biotechnol. Bioeng..

[135] Espinosa-Urgel M., Serrano L., Ramos J.L., Fernandez-Escamilla A.M. (2015). Engineering Biological Approaches for Detection of Toxic Compounds: A New Microbial Biosensor Based on the *Pseudomonas putida* TtgR Repressor. Mol. Biotechnol..

[136] Alibert S., Diarra J.N., Hernandez J., Stutzmann A., Fouad M., Boyer G., Pages J.M. (2017). Multidrug efflux pumps and their role in antibiotic and antiseptic resistance: A pharmacodynamic perspective. Expert Opin. Drug Met..

[137] Opperman T.J., Nguyen S. (2015). Recent advances toward a molecular mechanism of efflux pump inhibition. Front. Microbiol. Front. Microbiol..

[138] Jamshidi S., Sutton J.M., Rahman K.M. (2016). An overview of bacterial efflux pumps and computational approaches to study efflux pump inhibitors. Future Med. Chem..

[139] Kesherwani M., Michael Gromiha M., Fukui K., Velmurugan D. (2017). Identification of novel natural inhibitor for NorM—A multidrug and toxic compound extrusion transporter—An insilico molecular modeling and simulation studies. J. Biomol. Struct. Dyn..

[140] Kinana A.D., Vargiu A.V., May T., Nikaido H. (2016). Aminoacyl beta-naphthylamides as substrates and modulators of AcrB multidrug efflux pump. Proc. Natl. Acad. Sci. USA.

[141] Donner J., Reck M., Bunk B., Jarek M., App C.B., Meier-Kolthoff J.P., Overmann J., Muller R., Kirschning A., Wagner-Dobler I. (2017). The Biofilm Inhibitor Carolacton Enters Gram-Negative Cells: Studies Using a TolC-Deficient Strain of *Escherichia coli*. mSphere.

[142] Tintino S.R., Morais-Tintino C.D., Campina F.F., Costa M.D., Menezes I.R.A., de Matos Y.M.L.S., Calixto J.T., Pereira P.S., Siqueira J.P., Leal-Balbino T.C. (2017). Tannic acid affects the phenotype of *Staphylococcus aureus* resistant to tetracycline and erythromycin by inhibition of efflux pumps. Bioorganic Chem..

[143] Yang X., Goswami S., Gorityala B.K., Dornalaon R., Lyu Y.F., Kumar A., Zhanel G.G., Schweizer F. (2017). A Tobramycin Vector Enhances Synergy and Efficacy of Efflux Pump Inhibitors against Multidrug-Resistant Gram-Negative Bacteria. J. Med. Chem..

[144] Yang X., Domalaon R., Lyu Y., Zhanel G.G., Schweizer F. (2018). Tobramycin-Linked Efflux Pump Inhibitor Conjugates Synergize Fluoroquinolones, Rifampicin and Fosfomycin against Multidrug-Resistant *Pseudomonas aeruginosa*. J. Clin. Med..

[145] Adamson D.H., Krikstopaityte V., Coote P.J. (2015). Enhanced efficacy of putative efflux pump inhibitor/antibiotic combination treatments versus MDR strains of *Pseudomonas aeruginosa* in a Galleria mellonella in vivo infection model. J. Antimicrob. Chemother..

[146] Prasch S., Bucar F. (2015). Plant derived inhibitors of bacterial efflux pumps: An update. Phytochem. Rev..

[147] Shiu W.K., Malkinson J.P., Rahman M.M., Curry J., Stapleton P., Gunaratnam M., Neidle S., Mushtaq S., Warner M., Livermore D.M. (2013). A new plant-derived antibacterial is an inhibitor of efflux pumps in *Staphylococcus aureus*. Int. J. Antimicrob. Agents.

[148] Garvey M.I., Rahman M.M., Gibbons S., Piddock L.J. (2011). Medicinal plant extracts with efflux inhibitory activity against Gram-negative bacteria. Int. J. Antimicrob. Agents.

[149] Laudadio E., Cedraro N., Mangiaterra G., Citterio B., Mobbili G., Minnelli C., Bizzaro D., Biavasco F., Galeazzi R. (2019). Natural Alkaloid Berberine Activity against *Pseudomonas aeruginosa* MexXY-Mediated Aminoglycoside Resistance: In Silico and in Vitro Studies. J. Nat. Prod..

[150] Negi N., Prakash P., Gupta M.L., Mohapatra T.M. (2014). Possible Role of Curcumin as an Efflux Pump Inhibitor in Multi Drug Resistant Clinical Isolates of *Pseudomonas aeruginosa*. J. Clin. Diagn. Res..

[151] Li B., Yao Q., Pan X.C., Wang N., Zhang R., Li J., Ding G., Liu X., Wu C., Ran D. (2011). Artesunate enhances the antibacterial effect of {beta}-lactam antibiotics against *Escherichia coli* by increasing antibiotic accumulation via inhibition of the multidrug efflux pump system AcrAB-TolC. J. Antimicrob. Chemother..

[152] Muniz D.F., Dos Santos Barbosa C.R., de Menezes I.R.A., de Sousa E.O., Pereira R.L.S., Junior J.T.C., Pereira P.S., de Matos Y., da Costa R.H.S., de Morais Oliveira-Tintino C.D. (2021). In vitro and in silico inhibitory effects of synthetic and natural eugenol derivatives against the NorA efflux pump in *Staphylococcus Aureus*. Food Chem..

[153] Solnier J., Martin L., Bhakta S., Bucar F. (2020). Flavonoids as Novel Efflux Pump Inhibitors and Antimicrobials Against Both Environmental and Pathogenic Intracellular Mycobacterial Species. Molecules.

[154] Tariq A., Sana M., Shaheen A., Ismat F., Mahboob S., Rauf W., Mirza O., Iqbal M., Rahman M. (2019). Restraining the multidrug efflux transporter STY4874 of *Salmonella* Typhi by reserpine and plant extracts. Lett. Appl. Microbiol..

[155] Li J., Liu D., Tian X., Koseki S., Chen S., Ye X., Ding T. (2019). Novel antibacterial modalities against methicillin resistant *Staphylococcus aureus* derived from plants. Crit. Rev. Food Sci. Nutr..

[156] Karumathil D.P., Nair M.S., Gaffney J., Kollanoor-Johny A., Venkitanarayanan K. (2018). Trans-Cinnamaldehyde and Eugenol Increase *Acinetobacter baumannii* Sensitivity to Beta-Lactam Antibiotics. Front. Microbiol..

[157] Chambers C.S., Viktorová J., Řehořová K., Biedermann D., Turková L., Macek T., Křen V., Valentová K. (2020). Defying Multidrug Resistance! Modulation of Related Transporters by Flavonoids and Flavonolignans. J. Agric. Food Chem..

